# Successful Treatment of Cutaneous Scedosporium prolificans: An Emerging and Treatment-Resistant Fungal Pathogen

**DOI:** 10.7759/cureus.44738

**Published:** 2023-09-05

**Authors:** Lauren Flowers, Luke Witte, Susan M Zurowski

**Affiliations:** 1 Department of Dermatology, University of Missouri School of Medicine, Columbia, USA; 2 Department of Dermatology, University of Missouri, Columbia, USA

**Keywords:** cutaneous mycosis, antifungal drug resistance, itraconazole combined with minocycline, antifungal combination therapy, superficial dermatomycosis, cutaneous fungal infection, scedosporium prolificans

## Abstract

*Scedosporium prolificans* (*S. prolificans*) is an increasingly prevalent and treatment-resistant opportunistic fungus. The pathogen is known to cause a variety of clinical manifestations ranging from localized cutaneous disease to disseminated systemic infection. Herein we present an otherwise healthy 41-year-old male with biopsy-proven *S. prolificans* cutaneous infection. The patient experienced drastic clinical improvement on two months of a combination of oral itraconazole and oral minocycline. *S. prolificans* exhibits resistance to many antifungal agents, thus, single-agent antifungal therapy has a high failure rate and often results in the need for surgical excision or debridement. Recent accounts suggest that minocycline in combination with azole antifungals has a synergistic effect in treating *S. prolificans*. This case highlights the excellent response to combination oral therapies with minocycline and itraconazole. Prompt and efficacious treatment reduces the risk of destructive or disseminated disease and may avoid the need for surgical intervention.

## Introduction

*Scedosporium prolificans* (*S. prolificans*), formerly known as *Scedosporium inflatum*, is a ubiquitous fungus that was first described in 1984 and has recently emerged as a prominent opportunistic pathogen involved in invasive infections [1-3]. The species is categorized under the genus *Scedosporium*, with *S. prolificans* and *Scedosporium apiospermum* being the most clinically relevant pathogens within the genus [1]. *S. prolificans* is found in agricultural soil, potted plants, sewage, and polluted waters of temperate climates [1,2,4]. In the United States, *S. prolificans* is most prevalent in California and the southern states [1,2,4].

Both* S. prolificans *and *S. apiospermum* are known to cause a variety of clinical manifestations including colonization of the respiratory tract, localized deep subcutaneous or articular infections, and rapidly fatal disseminated disease [1]. The *Scedosporium *genus has recently been recognized as the most common cause of disseminated phaeohyphomycosis, however, it is often refractory to antifungal treatments resulting in high mortality in immunocompromised patients [2-5]. Infection with *S. prolificans* favors the immunocompromised, especially in disseminated disease [1,2,5]. However, immunocompetent individuals can be affected, and often present with keratitis, sinusitis, or subcutaneous soft tissue infections [1,2,5].

Isolated cutaneous infection with *S. prolificans *poses a treatment challenge of managing a pathogen that is increasingly resistant to first-line antifungal therapies [6]. Herein, we present an otherwise healthy patient with limited cutaneous *S. prolificans* infection that was treated successfully with a combination of antibiotic and antifungal therapy.

This case was previously presented as a poster presentation at the 2022 Missouri Dermatological Society Annual Meeting on September 16, 2022.

## Case presentation

**Figure 1:**
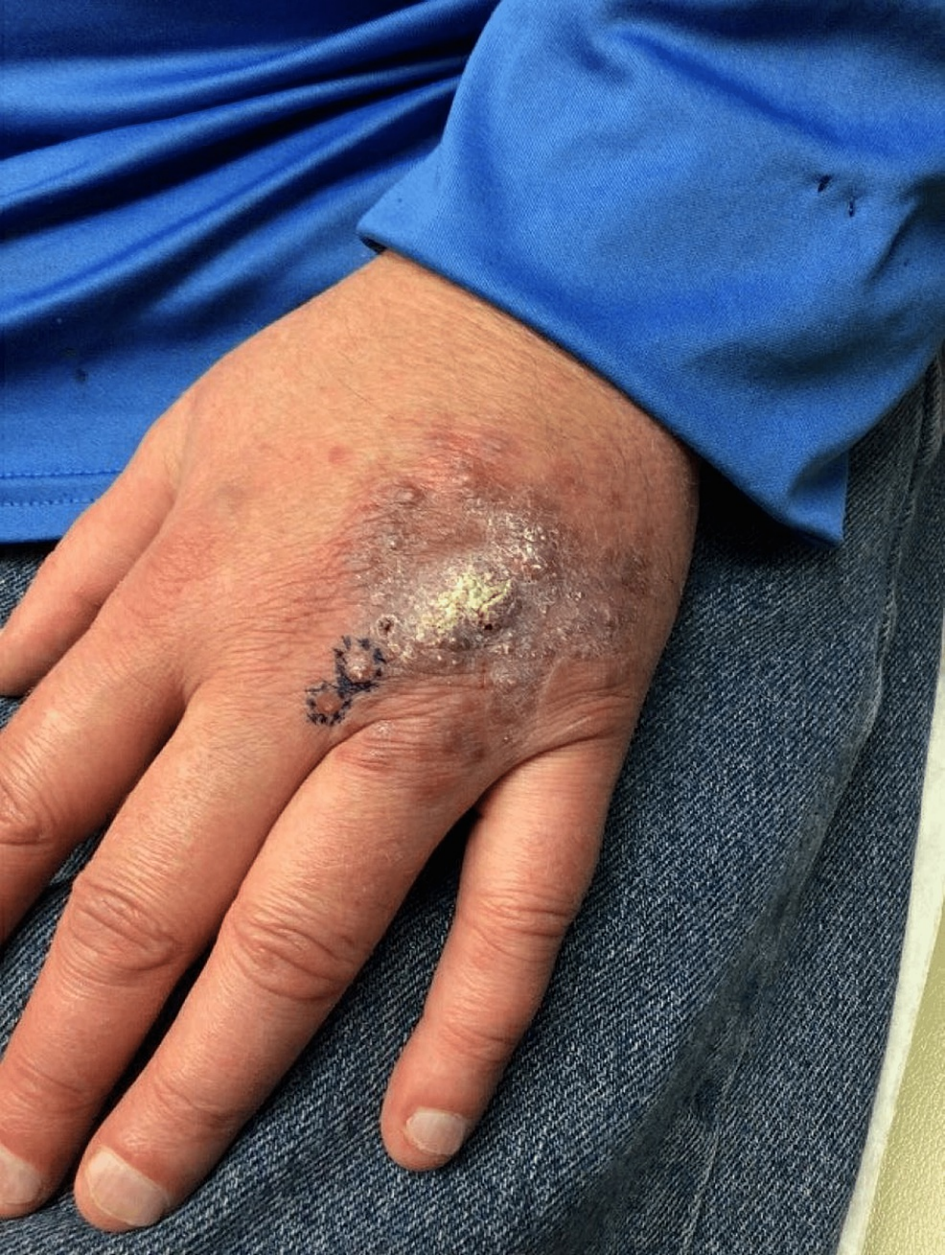
Clinical presentation of lesion on left dorsal hand of a 41-year-old male. Initial presenting exam of the left dorsal hand, significant for a 4 cm verrucous, hyperkeratotic, and indurated plaque with scattered erythematous papules along the periphery. Purple ink was used for the outline of punch biopsy locations.

We present a 41-year-old male who was seen in a dermatology clinic for two months for a non-healing, mildly pruritic wound over his left dorsal hand. The lesion first appeared after minor trauma while doing construction work and progressively increased in size. He initially saw his primary care physician (PCP) who prescribed a seven-day course of doxycycline. After this, the patient reported continued progression of the wound. He failed subsequent treatment by his PCP with topical triamcinolone 0.1% cream and oral sulfamethoxazole-trimethoprim. The patient had no significant past medical history or history of immunocompromising conditions such as diabetes mellitus, HIV, or autoimmune disease. Over the preceding two-month time period, the patient denied the presence of associated systemic symptoms such as fever, chills, chest pain, or shortness of breath. On physical exam, the patient was found to have a large 4 cm violaceous, indurated plaque with a white verrucous, hyperkeratotic center on the ulnar aspect of the left dorsal hand. The lesion was additionally surrounded by scattered folliculocentric firm papules (Figure 1).

The initial workup included two punch biopsies from the periphery of the lesion (circled with purple ink in Figure 1). One biopsy was obtained for histopathologic review and another for tissue culture. Histopathologic evaluation revealed granulomatous dermatitis with superficial pseudocarcinomatous hyperplasia. Additional diagnostic testing with periodic acid-schiff plus diastase, acid-fast bacilli, and Gram stains were negative for microorganisms, which raised suspicion for an atypical infection. The tissue culture resulted three weeks later with the isolation of *S. prolificans*.

**Figure 2:**
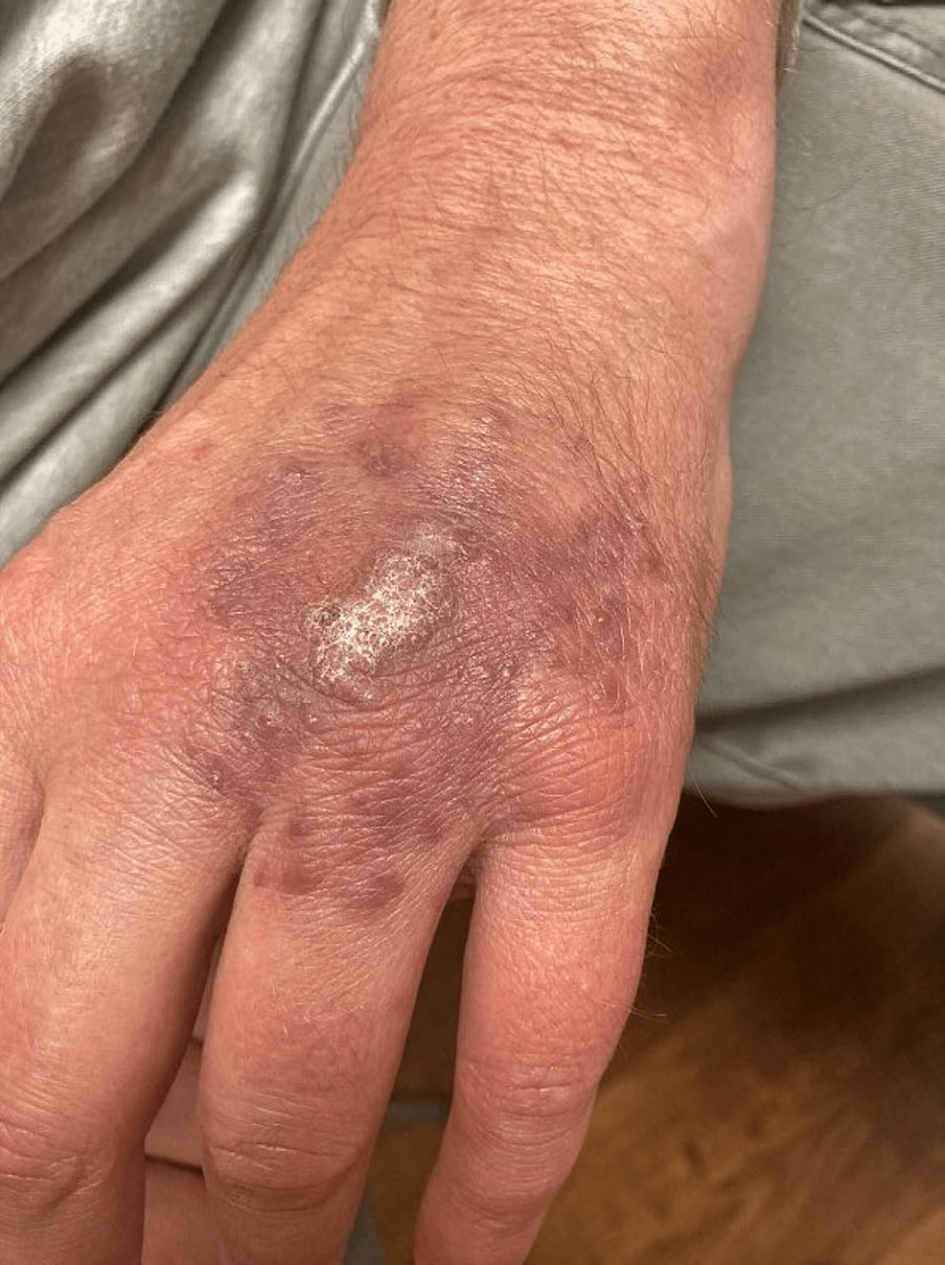
Clinical progression of cutaneous mycoses after two weeks of combined minocycline and itraconazole. Two weeks post-treatment with combined minocycline and itraconazole depicting dramatic improvement in cutaneous disease with a smaller central verrucous plaque and diminished surrounding hyperpigmented papules and macules.

**Figure 3:**
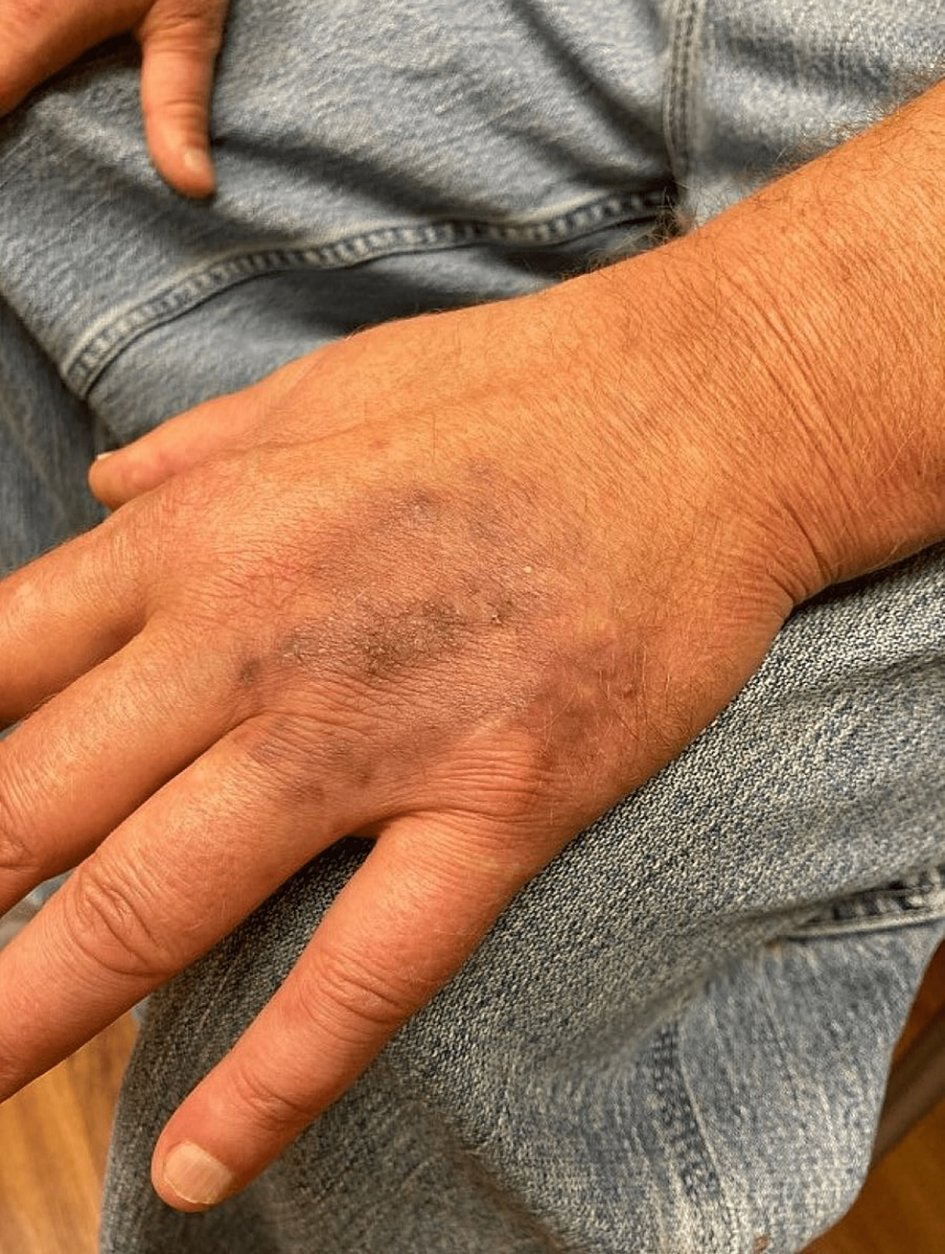
Clinical progression of cutaneous mycoses after six weeks of combined minocycline and itraconazole. Six weeks post-treatment with combined minocycline and itraconazole depicting substantial recovery with several remaining hyperpigmented macules coalescing into a circular patch. There are a few overlying white papules and a thinned central brown verrucous plaque.

While awaiting the culture results, the patient was empirically treated with oral minocycline 100 mg BID for one month but showed minimal clinical improvement. After culture results became available, oral itraconazole 200 mg BID was added to minocycline. The patient experienced dramatic improvement in cutaneous disease after just two weeks of combined itraconazole and minocycline therapy. He had significant thinning of the central verrucous plaque with surrounding residual hyperpigmented macules (Figure 2). In total, the patient completed two months of combined itraconazole and minocycline therapy and continued solo treatment with oral itraconazole 200 mg BID for one final month. At six weeks post combination treatment initiation, the patient had residual hyperpigmented macules with a central brown verrucous plaque (Figure 3), significantly improved from the initial presentation.

## Discussion

*S. prolificans *is an opportunistic and often recalcitrant pathogen known to cause a variety of clinical manifestations. Infection can cause either localized destructive disease or rapidly fatal hematogenous dissemination [1]. Clinical presentation includes four distinct conditions: superficial or subcutaneous dermatomycosis; respiratory tract infection; deep tissue infections including sinusitis, arthritis, or osteomyelitis; and disseminated disease [1,2]. Systemic involvement is most common in the immunocompromised patient and is associated with high mortality [1,2].

Cutaneous disease due to *Scedosporium *infection presents with progressive, destructive, and often indolent implantation of cutaneous mycosis known as a mycetoma [1,7,8]. This clinical presentation is similar to that of other cutaneous fungal infections, such as sporotrichosis [1,7,8]. Initial inoculation with *S. prolificans* generally occurs following traumatic insult and resultant direct contact with subcutaneous tissue [1]. The lower extremity is the most common location of disease, however, there have been reports of infection in the upper extremities or facial tissue [1]. The infected traumatic lesion is generally painless and increases in size slowly with the absence of associated constitutional symptoms [1,7,8]. Over time, the tissue may form progressively enlarging papules and nodules [1]. Ulcerations, sinus tracts, and fistulas may begin to form at greater than three months of infection [1]. Deep tissue and local articular infections occur with the progression of destructive lesions over time [1]. Mycetomas can result in significant tissue destruction and deformity if left untreated [1]. Notably, there has been one additional report in the literature of an isolated cutaneous infection with *S. prolificans* that presented similarly to this case, with superficial hyperkeratotic verrucous plaques [4].

Diagnosis of *S. prolificans* cutaneous infection is made under microscopic examination by utilizing a combination of histopathologic techniques as well as culture on routine fungal media [6]. Although histopathology alone cannot isolate *S. prolificans*, examination by traditional stains, such as the Gomori methenamine silver, hematoxylin and eosin, and periodic acid-Schiff, can highlight the presence of fungal elements, branching hyphae or fungal spores, allowing for presumptive diagnosis [1,6]. Although culture is often slow to result (taking two to ten days on average) it is critical for diagnosis, speciation, and susceptibility testing [1,4,6]. Molecular techniques for testing are being increasingly used to detect *Scedosporium* pathogens but are not currently standard-of-care for routine diagnosis [6]. Timely diagnosis is critical and allows for the early treatment of this highly resistant organism.

Infection with *Scedosporium *species is often difficult to treat due to high levels of resistance to most antifungal agents [1-3,8]. *Scedosporium *pathogens are inherently recalcitrant to common antifungals, and prior reports note concern for widespread resistance of *S. prolificans* to many antifungals including amphotericin B, flucytosine, ketoconazole, and fluconazole [1,4,6]. *S. prolificans* can additionally evade host defenses by forming aggregates with an external barrier matrix component [1,6]. Single-agent antifungal treatments have limited success due to high rates of resistance, often resulting in surgical excision or debridement for the management of refractory disease [1,2]. Occasional susceptibilities have been reported to mainly itraconazole and rarely miconazole [4]. Although, there have been limited studies on multi-modal treatment for cutaneous *Scedosporium *infection.

However, there are a number of accounts in the literature that point toward the increased effectiveness of combination antifungals in treating *S. prolificans*, including terbinafine with azole antifungals, as well as amphotericin B with different antifungals [2,3]. Most recently, Yang et al. (2022) suggested that minocycline in combination with azole antifungals has a synergistic effect in treating* S. prolificans*. In vitro and in vivo testing showed that minocycline in combination with itraconazole, voriconazole, and posaconazole was more effective against *Scedosporium *species than single antifungals alone [3]. Minocycline may provide antifungal properties related to its ability to lower cell surface hydrophobicity and reduce levels of 1,3-beta-D-glucan, which are important to fungal cell wall integrity [3].

This adds to the literature a case of successful treatment of cutaneous *S. prolificans* with a combination of minocycline and itraconazole. Further research is needed to understand the exact antifungal mechanism of minocycline and the long-term treatment outcomes.

## Conclusions

Our case illustrates a cutaneous infection with *S. prolificans* that had an excellent response to combination oral therapy, highlighting the proposed synergistic effects of minocycline with azole antifungals. As different fungal species, including *S. prolificans*, become increasingly resistant to current single antifungal agents, innovative treatment regimens are needed. Appropriate utilization of effective combination therapy regimens may improve clinical outcomes and mitigate progression to destructive disease, thus avoiding the need for surgical intervention. We strove to make other providers aware of emerging opportunistic infections, *S. prolificans*, and the potential need for combination therapies.
